# Recent Progress in Graphite Intercalation Compounds for Rechargeable Metal (Li, Na, K, Al)‐Ion Batteries

**DOI:** 10.1002/advs.201700146

**Published:** 2017-06-23

**Authors:** Jiantie Xu, Yuhai Dou, Zengxi Wei, Jianmin Ma, Yonghong Deng, Yutao Li, Huakun Liu, Shixue Dou

**Affiliations:** ^1^ Institute for Superconducting and Electronic Materials University of Wollongong Wollongong 2500 Australia; ^2^ School of Physics and Electronics Hunan University Changsha 410082 P. R. China; ^3^ Department of Materials Science and Engineering South University of Science and Technology of China 1088 Xueyuan Blvd Shenzhen 518055 P. R. China; ^4^ Materials Science and Engineering Program & Texas Materials Institute The University of Texas at Austin Austin TX 78712 USA

**Keywords:** Al ion batteries, graphite intercalation compounds, K ion batteries, Li ion batteries, Na ion batteries

## Abstract

Lithium‐ion batteries (LIBs) with higher energy density are very necessary to meet the increasing demand for devices with better performance. With the commercial success of lithiated graphite, other graphite intercalation compounds (GICs) have also been intensively reported, not only for LIBs, but also for other metal (Na, K, Al) ion batteries. In this Progress Report, we briefly review the application of GICs as anodes and cathodes in metal (Li, Na, K, Al) ion batteries. After a brief introduction on the development history of GICs, the electrochemistry of cationic GICs and anionic GICs is summarized. We further briefly summarize the use of cationic GICs and anionic GICs in alkali ion batteries and the use of anionic GICs in aluminium‐ion batteries. Finally, we reach some conclusions on the drawbacks, major progress, emerging challenges, and some perspectives on the development of GICs for metal (Li, Na, K, Al) ion batteries. Further development of GICs for metal (Li, Na, K, Al) ion batteries is not only a strong supplement to the commercialized success of lithiated‐graphite for LIBs, but also an effective strategy to develop diverse high‐energy batteries for stationary energy storage in the future.

## 1 Introduction

Since its discovery in 2004,^1^ graphene, consisting of a layer of *sp* ^2^ bonded carbon atoms arranged in a hexagonal or honeycomb lattice, has received strong attention. Owing to its high surface area (≈2630 m^2^ g^−1^), high thermal conductivity (≈5000 W mK^−1^), large charge carrier mobility (≈200 000 cm^2^ V^−1^ s^−1^), strong mechanical strength (≈130 GPa), and large Young's modulus (≈1 TPa), graphene holds great promise for practical applications in energy storage/conversion systems, such as supercapacitors (SCs), lithium ion batteries (LIBs), fuel cells, and solar cells.^2^ Graphite is a mineral with a layered structure, which is composed of many layers of graphene. Graphite intercalation compounds (GICs), with intercalated species between graphene layers, exhibit excellent physical and chemical properties comparable to those of pristine graphite. The physical and chemical properties of GICs are mainly related to the intercalant species, including alkali metal, metal oxides, metal chlorides, bromides, fluorides, oxyhalides, acidic oxides, and Lewis acids, as well as the quality of the graphene (e.g., lateral size, degree of exfoliation, conductivity, and defects).^3^ Since the concept of the GIC was published in 1841, the development of GICs has experienced several historical periods, leading to GICs with high conductivity, superconductivity (e.g., high transition temperature), and superb storage of hydrogen/lithium ions (**Table** **1**).^4^ There have been a large number of approaches developed for the mass production of GICs, as well as the exfoliation of high quality graphene using GICs.^5^ Most of these GICs have already been intensively reported for various applications in electrical/thermal conductors, catalysis, and energy storage.^6^, ^7^

**Table 1:** Short development history of GICs (updated from references^4^, ^7^) and their representative application in metal (Li, Na, K, Al) ion batteries

Year	Topics	Year	Topics
1841	H_2_SO_4_‐GIC (Schafhaut)^8^	1992	Rocking‐chair type intecalation (Guyomard)^9^
1926	K‐GIC (Cadenbach)^10^	1994	Dual intercalation molten salt (Carlin)^11^
1930	Graphite fluorides^12^	1996	“Dual‐carbon” secondary energy storage devices (McCullough)^13^
1932	FeCl_3_‐FUC (Thiele)^14^	1997	Electrochemistry of KC_8_ in lithium containing electrolyte (Tossici)^15^
1938	Dual‐carbon (Rüdorff)^16^	2000	“Dual‐Graphite” lithium ion batteries and GIC sodium ion batteries (Dahn)^17^
1964	K‐H‐GIC (Saeher)	2004	Potassium secondary cell (Eftekhari)^18^
1969	Daumas‐Herold mode (Daumas)^19^	2010	Potassium in graphite in KF melt (Liu)^20^
1972	High conductivity of GICs (Ubbelohde)^21^	2012	Dual‐ion batteries (Placke)^22^
1974	Li/(CF)_n_ primary batteries (Fukuda)^23^	2013	FeCl_3_‐GICs as anode for LIBs (Wang)^24^
1981	Ni(OH)_2‐_GICs secondary cell (Flandrois)	2014	Expanded graphite sodium ion batteries (Wen)^26^
	Ionic fluorine‐GICs (Nakajima)^25^		“Dual‐Graphite” batteries (Rothermel)^27^
1987	Metal chloride‐GICs by molten salts (Inagaki)^28^	2015	GIC secondary potassium batteries (Jian)^29^
			Al‐graphite batteries (Lin)^30^

As one of the most promising energy storage systems, LIBs have been widely used in portable and smart devices, owing to their high energy density, long cycling life, low cost, and environmental friendliness. Recently, LIBs have also been considered as one of most promising candidates for large‐scale application in electric vehicles (EVs) and grid‐scale energy storage. However, these practical applications require higher energy density.^31^ It is well known that the primary functional components of a LIB are the positive (cathode) and negative (anode) electrodes, the electrolyte, and the separator (membrane). The success of commercial LIBs is heavily dependent on the exploration of advanced electrode materials. Since the first prototype LIB cell was commercialized by the Sony Corporation in 1991, lithium intercalated graphite (LIG) as the anode for LIBs has always had great success in the commercial market owing to its low and flat working potential (close to Li^+^/Li), long cycle life, low cost, and environmental friendliness.^32^, ^33^, ^34^ Nevertheless, the low theoretical specific capacity of 372 mA h g^−1^ and poor Li‐ion transport rate (10^−12^–10^−14^ cm^2^ s^−1^) of LIG have limited its lithium storage performance in terms of energy and power densities and rate capability. Over the past two decades, a great number of anode materials, including modified LIG, carbonaceous materials, metal oxides, Li_4_Ti_5_O_12_, tin, and silicon, have been explored as alternative anodes for LIBs.^33^, ^35^ Some especially significant progress was achieved on a great number of carbonaceous materials,^36^, ^37^, ^38^ as well as modified LIG,^32^, ^39^ for LIBs with high theoretical capacities, high surface area, high conductivity, and superb chemical stability.^32^, ^36^, ^38^, ^39^ Similar to the case of LIG, there has also been recent progress on cationic GICs for other alkali‐ion batteries, including sodium‐ion batteries (SIBs), potassium‐ion batteries (PIBs), and aluminium‐ion batteries (AIBs). Apart from the use of GIC as anode in metal‐ion batteries, anion‐intercalated graphite compounds as cathodes for high‐performance alkaline (aluminium)‐ion batteries were also investigated over the past few years, although they were proposed quite early by McCullough (Table 1). It should be noted that, although some batteries have alkaline (e.g., Li and Na) ion containing electrolyte, with or without graphite anode, similar to some traditional LIBs or SIBs, these batteries (“dual‐ion” batteries) have a different ion storage mechanism to traditional LIBs For example, dual‐ion batteries (DIBs), as well as AIBs, are based on simultaneous intercalation of cations and anions into the cathode and anode, respectively. Consequently, we will briefly summarize the existing drawbacks, major progress, and emerging challenges for the development of ionic GICs as electrodes in the metal (Li, Na, K, Al) ion batteries, including traditional LIBs, SIBs, PIBs, and DIBs (including anionic‐type GICs for DIBs based on lithium and AIBs).

## 2 Structures of GICs

**Figure 1:**
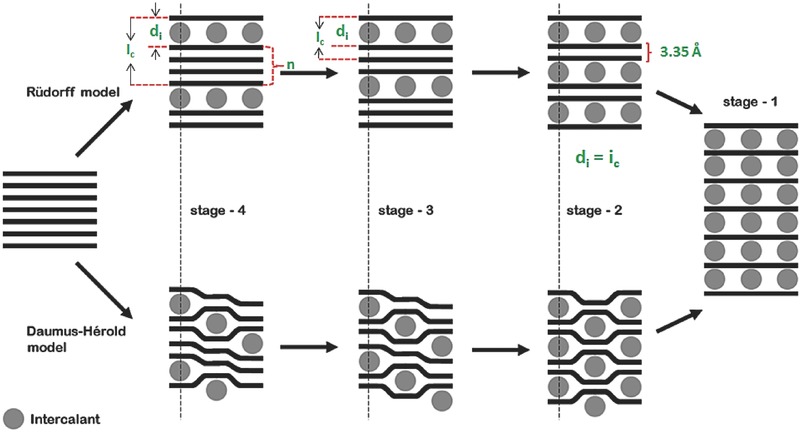
Schematic illustration of Rüdorff and Daumas–Hérold models (modified by reference^40^) for the staging mechanism of guest species intercalated into graphite. Reproduced with permission.^40^ Copyright 2014, Royal Society of Chemistry.

According to the character of their bonding (covalent and ionic), GICs can be generally classified into two categories: covalent GICs and ionic GICs.^41^ Covalent GICs include graphite oxide (GO), carbon monofluoride, and tetracarbon monofluoride. In contrast, ionic GICs include graphite salts (e.g., graphite nitrate, graphite bisulphate), graphite−alkali‐metal compounds, graphite‐halogen compounds, and graphite‐metal chloride compounds. Ionic GICs have received more attention than covalent GICs owing to the change in the electronic properties of graphite, which is ascribed to the π‐bonds in graphite that can accept/donate electrons from/to the intercalation, respectively. According to Rüdorff and Daumas–Hérold's models (**Figure** **1**), ionic GICs are further classified in terms of “staging”.^40^, ^42^ The stage (*n*) of GICs is determined by the number of graphene layers between two intercalant layers. For instance, in a stage‐1 GIC, each graphene sheet is separated from the others by intercalant galleries, while the stage‐2 GIC is composed of layers of two adjacent graphene sheets between intercalant galleries. The detailed gallery expansion of ionic GIC along the direction perpendicular (*c*‐axis) to the hexagonal plane of graphite (e.g. (002), Δ*d*) can be described as:^42^, ^43^

(1) $$\begin{array}{c} \Delta d=I_{c}-3.35\;Å\cdot n=d_{i}+3.35\;Å\;\;(n-2)\\ =l\cdot d_{\mathrm{obs}}-3.35\;Å\cdot n \end{array}$$

where *I*_c_, *d*_i,_ *d*_obs_, and l are the periodic repeat distance, the intercalant gallery height_,_ the observed value of the spacing between two adjacent planes, and the index of (00*l*) planes oriented in the stacking direction, respectively.

## 3 Electrochemistry of GICs

Several electrochemical strategies have been widely used to prepare GICs as electrodes for energy storage systems (e.g., supercapacitor and metal‐ion batteries, as shown in Table 1) over past decades. In an electrochemical system, ionic GICs (CX_m_), are formed by insertion (intercalation) of chemical species between the layers of graphene. The reaction can be simply expressed as:

(2) $$C+mX\to {\mathrm{CX}}_{m}$$

where the interaction between host (graphite) and the guest (X) is a reversible redox process. If the insertion of X into graphite is an anion (X^m−^), the reversible reaction is expressed as:

(3) $$C_{x}+X↔C_{x}{}^{m+}\cdot X^{m-}$$

Because X accepts *m* electrons from the π‐electron carbon network, the GIC is regarded as an “acceptor‐type” GIC. In contrast, if X donates an electron to the carbon network, the GIC is a “donor‐type GIC” with cation insertion (X^m+^). The reversible reaction can be expressed as:

(4) $$C_{x}+X↔C_{x}{}^{m-}\cdot X^{m+}$$

Therefore, ionic GICs can be further divided into cationic (donor‐type) and anionic (acceptor‐type) GICs based on the insertion of ion types (cation and anion).

## 4 GICs for Metal (Li, Na, K, Al) Ion Batteries

The intercalation chemistry of GICs is extremely rich in terms of various kinds of intercalants (X). Among all types of X, the alkali elements, group 1 of the Periodic Table (group IA), are very reactive and find it easy to lose their outermost electrons to become cations with charge. This helps alkali atoms to form ionic bonds with other elements. In addition, the alkali elements in group 1 are similar to each other. Therefore, many efforts have been devoted to exploring alkali based GICs. There has been a long development history of cationic GICs with alkali ions (X = Li, Na, K) as species intercalated into graphite, including the chemical formation of Na(NH_3_)_2_C_12_, Li(HMPA)C_32_, and Na(HMPA)C_27_,^44^ where HMPA = hexamethylphosphoramide, and the electrochemical formation of Li*_x_*C_6_, Na*_x_*C_6_, KC_6_, and C–X with various C patterns (e.g., one‐dimensional (1D) carbon nanotube (CNT), two‐dimensional (2D) graphene, three‐dimensional (3D) carbon, and amorphous carbon).^45^ Unlike cationic‐type GICs intercalated by alkali ions (X = Li, Na, K) that are used as anodes in electrochemical systems, the intercalation of GICs with anions from alkali‐based electrolyte taking place on the cathode side have also been considered recently.

### 4.1 Cationic‐type GICs for LIBs

**Figure 2:**
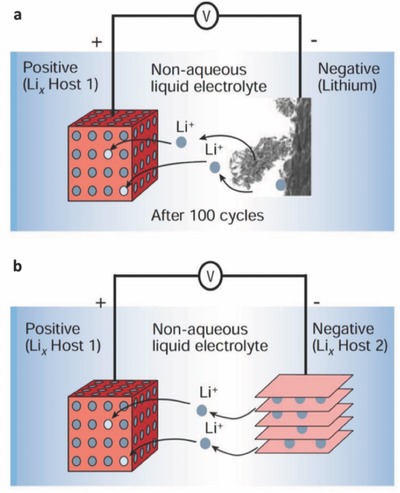
Schematic illustration of operating principles of LIBs with anode of (a) lithium metal (lithium primary batteries) and (b) graphite. Reproduced with permission.^46^ Copyright 2001, Nature Publishing Group.

**Figure 3:**
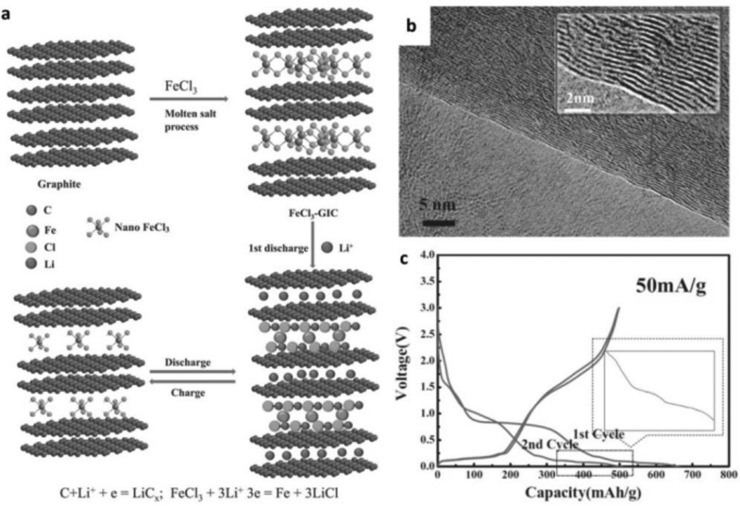
Schematic illustration of the lithiation‐delithiation mechanism, (b) high resolution transmission electron microscope (TEM) image (higher magnification in inset), and (c) discharge‐charge profiles at a current density of 50 mA g^−1^ in the voltage range of 0–3 V of FeCl_3_‐GIC. Reproduced with permission.^24^

As one representative carbon material, graphite has been successfully used as an anode material for LIBs. From the mechanism of lithium intercalation into graphite, lithium ions mainly diffuse in the in‐plane direction and then occupy the sites between two adjacent graphene planes during the lithium intercalation process. Each lithium occupies the center of a hexagonal carbon (C) ring with a Li‐C_6_‐Li‐C_6_ sequence along the *c*‐axis in the fully lithiated state,^47^ delivering a theoretical capacity of 372 mAh g^−1^ (**Figure** **2**).^46^ Nevertheless, the structure of graphite, including the strong *sp* ^2^ hybridized C—C bonds of graphene and the dense stacking of hexagonal graphene sheets bonded by van der Waals interactions, results in a poor rate capability during the lithiation‐delithiation process, especially the liathiation process.^48^ The relatively low theoretical capacity and poor rate capability of lithium intercalated graphite are insufficient to provide very high energy/power densities. Over past decades, a great number of carbonaceous materials with high theoretical capacities, as well as exceptional properties (e.g., high surface area, high conductivity, excellent chemical stability), have thus been explored as anode materials for LIBs, including 1D carbon nanotubes, carbon nanowires, carbon nanofibers,^49^ 2D functional graphene nanosheets,^36^, ^50^ and other hybrid structures (e.g., amorphous, porous carbon).^51^ Nevertheless, graphite still preserves its advantages as anode for LIBs compared to those carbonaceous materials, including relatively low surface area, low volume expansion, high initial Coulombic efficiency (CE), and rich natural resources. Although a high surface area for carbonaceous materials, especially high prismatic or non‐basal‐plane graphite surfaces, is beneficial for the quick penetration of electrolyte, rapid diffusion of ions, and large amounts of ion storage, it will also cause more electrolyte decomposition and the formation of a denser solid‐electrolyte interphase (SEI) layer, leading to a poor initial CE. Therefore, a moderate surface area for carbonaceous materials is still preferred.^22^, ^52^ Recently, a FeCl_3_‐GIC prepared by melting FeCl_3_ into graphite delivered a high reversible capacity of 500 mAh g^−1^ with 100% capacity retention after 400 cycles (**Figure** **3**).^24^ The excellent performance of the FeCl_3_‐GIC was attributed to the stable structure of the GIC, which effectively accommodated the volume changes upon lithiation and delithiation. The limited initial CE of 76% for FeCl_3_‐GICs as anode for LIBs still needs to be further improved, however. In addition, stage‐2 and stage‐1 FeCl_3_‐GICs with improved performance were also prepared by a hydrothermal method^53^ and an ultrasonication method.^54^ These results once again indicate the advantages of GICs with stable structure, as well as high electronic conductivity and increased surface area of intercalated GICs.

### 4.2 Cationic‐type GICs for SIBs

The great success of lithiated graphite and the search for novel alternatives to lithiated GICs have spurred the rapid growth of SIBs recently. Compared to Li, Na has higher natural abundance, lower cost, and similar chemical/physical properties. The detailed natural resources, and the physical and chemical properties of Li and Na are compared in **Table** **2**. In contrast to the theoretical capacity of graphite for lithium ion intercalation, there is only a small amount of Na that can be stored in graphite, with a reversible capacity of less than 35 mAh g^−1^ (∼NaC_64_).^55^ This is mainly attributed to the unfavorable mismatch between the graphite structure and the size of the Na ion.^56^ Therefore, to enlarge the interlayer spacing of graphite is critically important for Na ion intercalation.^57^

**Table 2:** Comparison of characteristics of representative metals for non‐aqueous rechargeable batteries

Element	Lithium (Li)	Sodium (Na)	Potassium (K)
Date of discovery	1817	1807	1807
Abundance of elements in Earth's crust (rank)	33^th^	6^th^	7^th^
Density @ 293 K (g cm^−3^)	0.53	0.97	0.86
Relative atomic mass	6.94	22.98	39.10
Ionic radius (A^+^/Å)	0.76	1.02	1.38
Melting point (°C)	180.5	97.7	63.7
*E*° (V) vs. SHEa)	−3.04	−2.71	−2.93
*E*° (V) vs. Li^+^/Li	0	0.33	0.11

^a)^ SHE: Standard hydrogen electrode.

**Figure 4:**
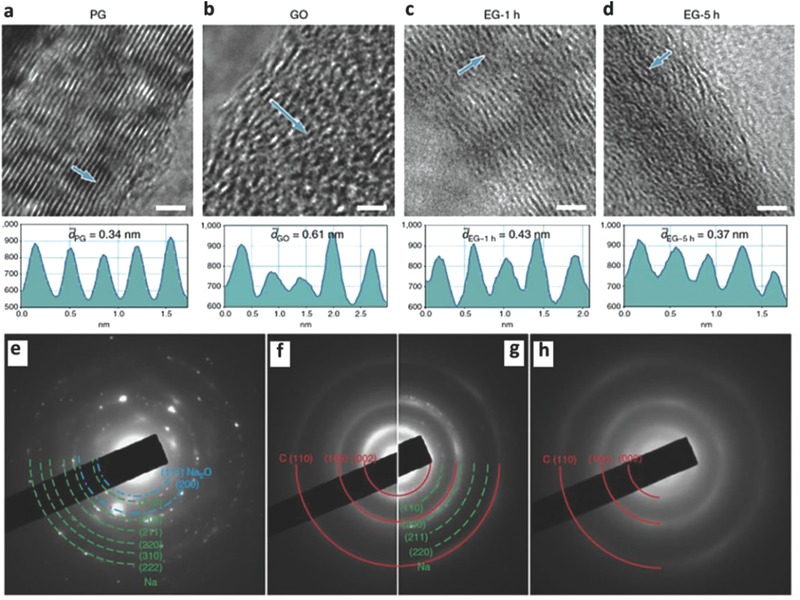
High‐resolution TEM images of (a) PG (pristine graphite), (b) GO, (c) EG‐1h and (d) EG‐5h. Scale bars: 2 nm. Contrast profiles along the arrows indicate the interlayer spacings of the corresponding samples. In situ TEM investigation of sodium storage mechanism in EG‐1h at different states (a) pristine state, (j) after the first sodiation, and (k) after the first desodiation. (e–h) Corresponding selected area electron diffraction (SAED) patterns of Figure 4. a–d Reproduced with permission.^26^ Copyright 2014, Nature Publishing Group.

To expand graphite with an enlarged interlayer lattice distance and a long‐range‐ordered layered structure a modified Hummers' method was employed. The expanded graphite was synthesized by oxidizing pristine graphite to graphite oxide, followed by heat treatment to partially reduce the graphite oxide. The interlayer spacing of expanded graphite was effectively regulated by the duration of chemical oxidation and reduction processes (**Figure** **4**a–d).^26^ As a result, the expanded graphite with an enlarged interlayer lattice distance of 4.3 Å delivered a reversible capacity of 284 mAh  g^−1^ at 20 mA g^−1^. At a higher current density of 100 mA g^−1^, it displayed a reversible capacity of 184 mAh g^−1^ at 100 mA g^−1^, and retained 73.9% of its capacity after 2000 cycles. The excellent cycling stability was attributed to its stable structure, similar to that of graphite, during the process of Na^+^ insertion and extraction, as evidenced by in situ transmission electron microscopy (TEM) (Figure 4a–d) and corresponding selected area electron diffraction (SAED) patterns (Figure 4e–h). On the other hand, recent investigations on co‐intercalation with suitable electrolyte solvents also show an insightful understanding of graphite as anode material for high energy SIBs.^56^, ^58^, ^59^, ^60^ For example, when ether‐based electrolytes (tetraethylene glycol dimethyl ether (TEGDME)) with a high donor number were used, natural graphite exhibited superb Na^+^‐solvent co‐intercalation combined with pseudocapacitive behaviour, leading to a high rate capability (100 mAh g^−1^ at 5 A g^−1^) and long‐term cycling stability of 2500 cycles.^59^ The remarkable intercalation pseudocapacitive behaviour was also observed in graphite for SIBs with the use of a linear ether‐based (tetraglyme (TGM)) electrolyte, which exhibited improved rate capability (110 mAh g^−1^ at 10 A g^−1^) and cycling stability (6000 cycles).^60^ Such formation of ternary graphite intercalation compounds (t‐GIPs) through the stage‐evolution process was further confirmed by ex situ X‐ray diffraction (XRD), in situ Raman spectroscopy, and voltammetry analysis.^60^ The superior cycling stability once again indicates the stable structure and high electrical conductivity of GICs. Apart from graphite, numerous carbonaceous materials (from 1D to 3D) with high theoretical capacities have been also identified as anodes for SIBs. Compared to their LIB counterparts, however, these carbon‐based anodes suffer from very low observed capacities, low initial CEs, and/or rate capabilities.^57^, ^61^

### 4.3 Cationic‐type GICs for PIBs

**Figure 5:**
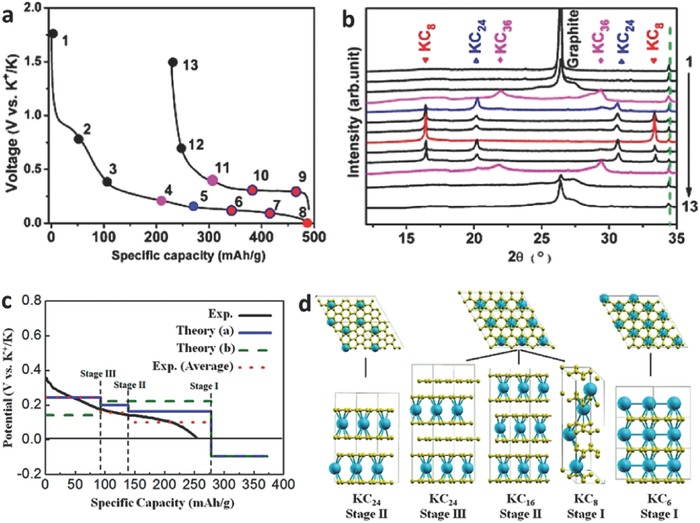
a) Initial discharge‐charge profile of graphite at C/10. b) Ex situ XRD patterns of electrodes at different voltage steps (marked in Figure 5. a). c) Calculated potential profile for K ion intercalation into graphite, and d) Scheme of the different stages of K‐intercalated graphite; Blue: K, Yellow: C. a–b) Reproduced with permission.^29^ and c–d) Reproduced with permission.^65^ Copyright 2015, American Chemical Society.

Along with the rapid development of LIBs and SIBs, PIBs have also become an attractive alternative to LIBs or SIBs, owing to the abundant natural resources and similar chemical/physical properties of potassium (K) to Li or Na (Table 2). Despite an early study on the structures and chemical/physical properties of K‐based GICs in the 1930s,^62^ the electrochemical intercalation of potassium ions into graphite in KIBs attracted less attention than LIBs or even SIBs in the same period.^56^, ^58^, ^59^, ^60^ Tossici et al.^15^ employed a fully intercalated K‐GIC (KC8) as the anode in a LIB, and found that the K ions were successfully extracted out of KC8 during the charge process. During the following discharge process, however, Li ions are inserted into graphite to form lithiated GICs. Liu et al.^20^ found that the electrochemically reversible intercalation of potassium into graphite can occur in molten salt of KF at the high temperature of 1163 K. The unstable and severe damage to the resulting K‐GIC structure, however, as well as the high operating temperature, posed huge challenges for the development of KIBs.^20^, ^63^ Along with the pioneering work on the use of Prussian blue as the cathode for KIBs^18^ and non‐graphitic carbon nanofibers as anode for KIBs,^64^ a room‐temperature electrochemical KIB system employing potassium insertion/extraction into/from graphite in a non‐aqueous electrolyte of 0.8 m KPF_6_ solvated in ethylene carbonate (EC)/diethyl carbonate (DEC) was reported.^29^ The reversibility of graphite and potassiated graphite (stage‐III KC36, stage‐II KC24, and stage‐IKC8) during the potassiation‐depotassiation process was confirmed by the ex situ XRD technique (**Figure** **5**a–b). This result is also consistent with the theoretical results for different stages of potassium intercalation into graphite (Figure 5c–d).^65^ At present, the limited electrochemical performance (e.g., cycling stability, cycling life, and rate capability) and volume expansion (up to 61%) of GIC remains a challenge for using graphite anode for KIBs.^29^ To solve these critical issues, many efforts have been made towards the exploration of carbonaceous materials with high surface area and rich porosity instead of graphite,^66^, ^67^ and efficient binders and electrolytes for KIBs.^67^, ^68^ Compared to LIBs or even SIBs, the history of the electrochemistry of PIBs is generally short. As expected, the successful strategies for the development of LIBs or SIBs are available for PIBs.

### 4.4 Anionic‐type GICs for DIBs Based on Lithium

**Figure 6:**
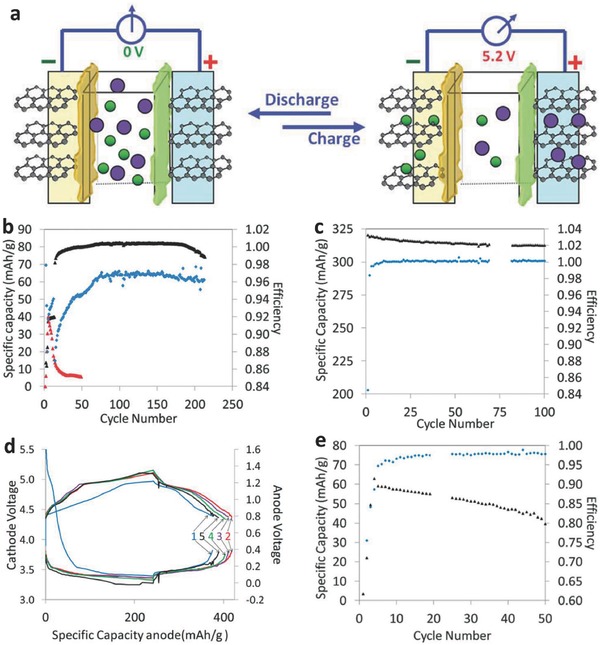
a) Schematic illustration of a dual‐graphite intercalation full cell. b) Cycling performance of the Li/MCMB 10–28 (MCMB 10–28 graphite from Osaka Gas) half‐cell at C/14 in a fluoroethylene carbonate (FEC) based electrolyte: Capacity (black) and CE (blue), and a 1.2 M LiPF_6_ EC–ethyl‐methyl carbonate (EMC) electrolyte: Capacity (blue). c) Cycling performance of half‐cell Li/CGP‐G5 (CGP‐G5 graphite from Conoco Phillips) at C/20. d) Discharge‐charge profiles of half‐cell CGP‐G5 anode (bottom) and MCMB 10–28 cathode (top) for five cycles. e) Cycling performance of ‘dual‐graphite' full cell: Specific capacity (black) and CE (blue). Reproduced with permission.^[69]^ Copyright 2014, Royal Society of Chemistry.

As discussed in sections 4.1–4.3, the graphite takes up alkali ions from the electrolyte (cathode) into its layer host structure. The resultant cationic GIC is called “donor‐type” graphite. While the “donor‐type GIC” is commonly demonstrated in the field of LIBs, SIBs, or PIBs, “acceptor‐type” GICs were suggested in the “dual‐carbon” cells. McCullough et al.^13^, ^70^ first proposed a rechargeable “dual‐carbon” cell in patents using pyrolyzed polyacrylonitrile (PAN) electrodes as both cathode and anode, with LiClO_4_ or LiPF_6_ salts in a propylene carbonate (PC) solvent as the electrolyte. The pyrolyzed PAN and the PC electrolyte are not suitable for anion intercalation, however, because of the disorder of the pyrolyzed PAN and easy exfoliation of graphite. Carlin et al. investigated the reductive and oxidative intercalation of ions into graphite in a single‐graphite‐electrode cell^71^ and two‐graphite‐electrodes cell.^11^ It was found that the cell with two graphitic electrodes in the electrolyte (DMPI^+^/AlCl_4_^−^) displayed an open circuit voltage of 3.5 V with a cycling efficiency of 85%.^11^ Dahn et al.^17^ exhibited a dual‐graphite cell with simultaneous intercalation at both the positive anode and the negative cathode in conventional electrolytes, namely 2 m LiPF_6_ in ethyl methanesulfonate (EMS) and 3 m LiPF_6_ in EC/DEC. The cell displayed a capacity of 140 mAh g^−1^ at a high potential of up to 5.5 V vs. Li^+^/Li. The oxidation stability of routine electrolyte at high voltage is a critical issue, however. Novak's group^72^ found that the PF_6_^−^ anion intercalation in graphite occurs at higher than 4.8 V in various electrolytes, including EMS, PC, EC, dimethyl carbonate (DMC), and ethyl‐methyl carbonate (EMC). Moreover, both the energy density and the specific capacity of dual graphite cells are largely reliant on the concentration and molecular weight of the electrolyte salt due to the changes in the viscosity and ionic conductivity of the electrolyte during the charge‐discharge process,^17^ as well as the upper cut‐off potential, the average discharge voltage of the cell, and the anion size.^73^ In order to improve the electrochemical stability window of both the electrolyte solvent and the anion, Winter's group^73^, ^74^ employed an ionic liquid electrolyte consisting of 1 m lithium bis(trifluoromethanesulfonyl)imide (LiTFSI) in *N*‐butyl‐*N*‐methylpyrrolidinium bis(trifluoromethanesulfonyl)imide (Pyr_14_TFSI) in a dual‐ion battery (DIB) with graphite as cathode and Li metal as anode. The fabricated cell delivered excellent cycling stability and capacity retention above 99% (≈50 mAh g^−1^) after 500 cycles. Unlike conventional “rock‐chair” LIBs, in which the electrolyte was mainly considered as the charge carrier with negligible participation in the intercalation reactions, the “dual‐carbon or dual‐graphite” batteries were called “dual‐ion” batteries by Winter's group.^73^, ^74^ Very recently, a 5.2 V “dual‐graphite or dual‐ion” battery using a high voltage electrolyte based on fluorinated solvent, LiPF_6_ in fluoroethylene carbonate (FEC)/methyl ethyl carbonate (EMC) was fabricated with an additive of 5 mM tris(hexafluoro‐isopropyl) phosphate (HFIP) was fabricated. As shown in **Figure** **6**a, the simultaneous intercalation of PF_6_^−^ anions and Li^+^ cations both take place at graphitic electrodes. Both the half “dual‐graphite” cells using the optimized electrolyte with the HFIP additive displayed relatively stable cycling for hundreds of cycles (Figure 6b–d).^69^ The cycling stability of the full “dual‐graphite” cell was limited, however, with only ≈70% capacity retention after 50 cycles compared to the graphitic half‐cell (Figure 6e). In order to obtain a feasible “dual‐graphite” cell, many efforts have been devoted to developing efficient electrolytes (e.g., anion and solvents) and alternative batteries (e.g. aluminium, sodium, and potassium based ion batteries) to lithium‐based dual‐graphite batteries.^75^, ^76^, ^77^

A number of electrolytes, including different types of anions (e.g. PF_6_^−^, BF_4_^−^, TFSI^−^, ClO_4_^−^, CF_3_SO_3_^−^, (CF_3_SO_2_)_2_N^−^, and other fluoride ions) and solvents (e.g. EC, DEC, DMC, EMC, dimethyl sulfoxide (DMSO), dimethylformamide (DMF), ethyl‐methyl sulfone (EMS), and Pyr_14_TFSI) have been used to investigate the electrochemical anion intercalation into graphite‐based cathode.^78^, ^79^, ^80^ Conventional commercial electrolyte solvents (e.g., EC, DEC, EMC, and DMC) mainly have limitations to their oxidation stability (<4.6 V, vs. Li^+^/Li).^81^ The solvents PC, DMSO, DMF, EMS, or Pyr_14_TFSI result in exfoliation and destruction of the graphite anode lattice, leading to deterioration in the electrochemical performance. Recently, it was found that the additive of ethylene sulfite (ES) in Pyr_14_TFSI based ionic liquids (ILs) enabled the formation of a stable SEI and thus stabilized both graphitic electrodes with a reversible capacity of about 50 mA h g^−1^ for 500 cycles.^82^ Very recently, a series of pyrrolidinium‐based ILs were systematically studied to find their effects on electrochemical anion intercalation behaviour into graphite. The onset potential using the imide‐based ILs is in the order of bis(pentafluoroethanesulfonyl)imide (BETI) > bis(fluorosulfonyl)imide (FSI) > fluorosulfonyl‐(trifluoromethanesulfonyl)imide (FTFSI) > FSI/TFSI > TFSI > TFSI/FSI).^79^ Compared to conventional organic electrolytes, ILs are promising candidates for high‐voltage anionic graphite batteries because of their non‐volatility, non‐flammability, high thermal and chemical stability, and wide electrochemical window.^33^ They always have high viscosity (e.g., vs. conventional carbonate based electrolyte), however, which induces low ionic conductivity and poor wettability of the separator and electrode materials. Moreover, the high price of ILs is another issue that should also be addressed in the future. In addition, the presence of side reactions within the graphitic electrode, which result from surface groups and anion intercalation at high voltage, may also degrade the electrochemical performance of the cell because of the electrolyte decomposition inside the carbon and the degradation of the graphitic structure.^76^, ^83^ Optimized thermal treatment of carbon may be a promising solution.

**Figure 7:**
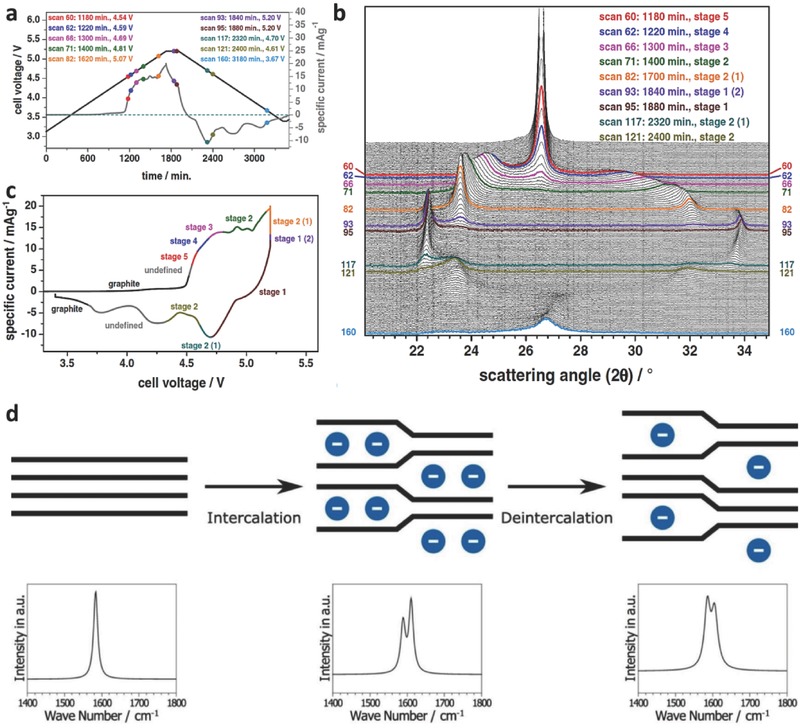
a) *U*‐*t* curve and (b) the corresponding in situ XRD patterns of FTFSI^−^ intercalated graphite, and (c) assigned stages in different voltage regions. d) Schematic representation of the stages of GICs (top) and the corresponding Raman spectra (bottom). (a–c) Reproduced with permission^80^ and (d) reproduced with permission.^84^ Copyright 2016, Elsevier.

In the development of anionic GICs for LIBs, characterization techniques such as XRD, Raman spectroscopy, X‐ray photoelectron spectroscopy (XPS), and cyclic voltammetry, as well as computational simulations, have been widely employed. Owing to the intercalant layer spacing (*d*_i_), enlarged by the intercalation compounds, XRD has been used to characterize the staging mechanism and the amount of expansion of the graphite structure. Compared to ex situ techniques, in situ techniques are more accurate for revealing the electrochemical activity and dynamics of electrode materials in an electrochemical system.^80^, ^85^ **Figure** **7**a–c shows in situ XRD patterns that can be used to identify the stage numbers of the GICs which result from the insertion and extraction of FTFSI^−^ anions at different voltages. As shown in Figure 7b, the in situ XRD results clearly indicate that the insertion and extraction of FTFSI^−^ anions into/from graphite are asymmetrical processes, as evidenced by the residual stage 1 GIC after the complete cathodic reaction, though excellent cycling stability of the cell was reported for hundreds of cycles.^80^, ^82^ A certain amount of TFSI^−^ anions within the GICs after the full de‐intercalation process was also confirmed by in situ Raman spectroscopy.^84^ As shown in Figure 7d, the non‐split G band of the stage l GIC or graphite cannot be recovered from the GIC at the end of the de‐intercalation process, which still shows remanent splitting of the G band due to the intercalation. In addition to XRD and Raman techniques, various other characterization techniques, such as photoelectron spectroscopy, dilatometry, and cyclic voltammetry, as well as computational simulations, have also been performed to further determine the *d*_i_, molecular orientation, and chemical composition of the resulting GIC cathode.^30^, ^86^

### 4.5 Anionic‐type GICs for AIBs

**Figure 8:**
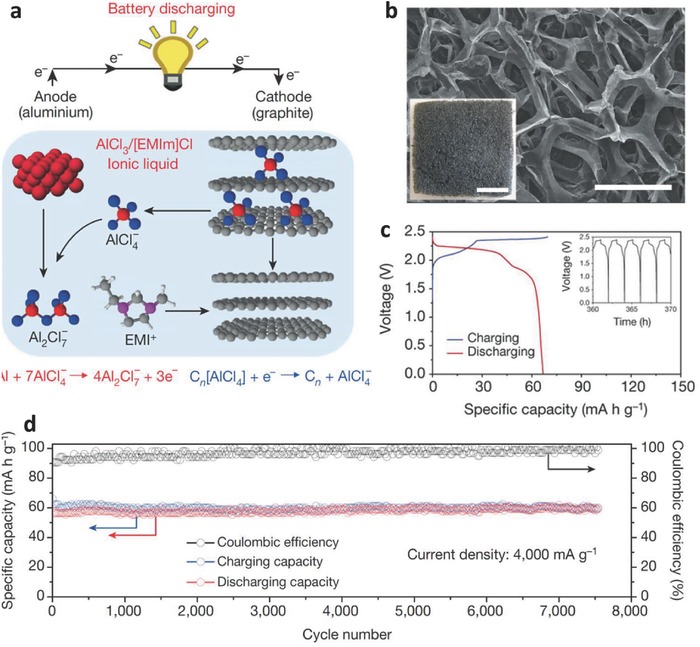
a) Schematic representation of rechargeable Al‐graphite cell. b) SEM image of graphitic cathode scale bar: 300 µm; inset, photograph of graphitic foam, scale bar: 1cm. c) Discharge‐charge curves of Al‐graphite cell at a current density of 66 mA g^−1^; inset: charge and discharge cycles over time. d) Long‐term cycling performance of the cell over 7500 cycles at a current density of 4 A g^−1^. Reproduced with permission.^30^ Copyright 2015, Nature Publishing Group.

Despite the advantages of greater abundance and lower cost than the alkali elements (Table 2), higher theoretical energy density (three valence electrons vs. one for alkali metals), and safer characteristics, aluminium was not widely studied for AIBs until 2015.^87^ This is mainly due to the low working voltage, lack of a distinct working plateau, and poor cycling life of AIBs. A breakthrough work from Dai's group^30^ has helped the AIBs to recapture great attention as a promising energy storage system. As shown in **Figure** **8**a, the AIB was constructed from an aluminium foil anode, a graphitic foam cathode (Figure 8b), and an ionic liquid electrolyte based on AlCl_3_/1‐ethyl‐3‐methylimidazolium chloride. The basic working principle of AIBs can be simply expressed as:

(5) $$\mathrm{Anode}:\;\;4{\mathrm{Al}}_{2}{\mathrm{Cl}}_{7}{}^{-}+3e^{-}↔\mathrm{Al}+7{\mathrm{AlCl}}_{4}{}^{-}$$

(6) $$\mathrm{Cathode}:\;C_{n}+{\mathrm{AlCl}}_{4}{}^{-}↔C_{n}\left[{\mathrm{AlCl}}_{4}\right]+e^{-}$$

(7) $$\begin{array}{c} \mathrm{Total}:\;\;4{\mathrm{Al}}_{2}{\mathrm{Cl}}_{7}{}^{-}+3C_{n}+3{\mathrm{AlCl}}_{4}{}^{-}\\ ↔3C_{n}\left[{\mathrm{AlCl}}_{4}\right]+\mathrm{Al}+7{\mathrm{AlCl}}_{4}{}^{-} \end{array}$$

where *n* is the molar ratio of carbon atoms to intercalated anions in the graphite.

**Figure 9:**
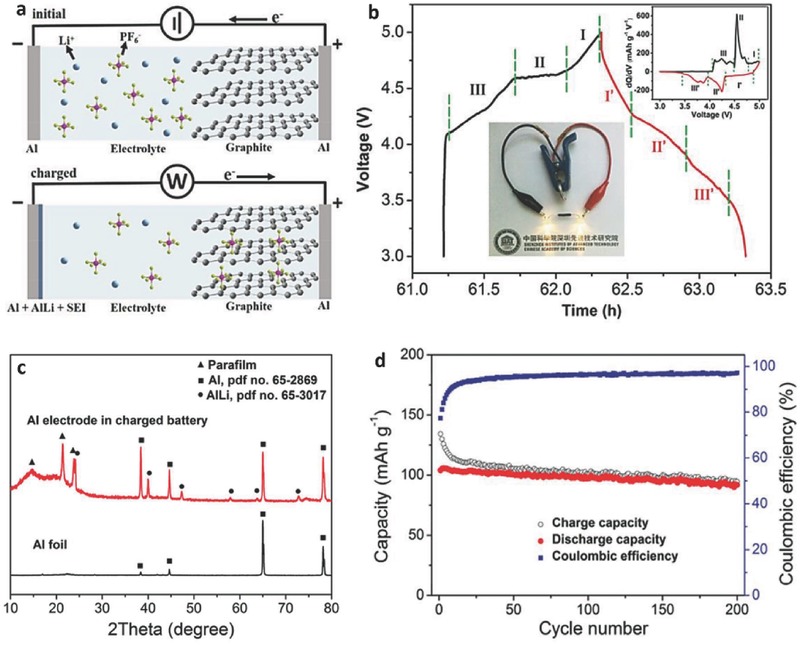
a) Schematic illustration of the Al‐graphite cell in the initial state (upper) and the charged state (lower). b) Discharge‐charge profile of the cell at 0.5 A g^−1^. Insets are the *dQ*/*dV* differential curve and a photograph of a light emitting diode (LED) charged by the Al‐graphite cell. c) XRD patterns of a fresh Al and a charged Al electrode. d) Long term cycling performance of the Al‐graphite cell at 0.2 A g^−1^. Reproduced with permission.^89^

The AIB with this working principle showed a working voltage plateau at 2 V, a reversible specific capacity of 70 mAh g^−1^ with a CE of 98% at a current density of 66 mA g^−1^ (Figure 8c), and maintained ≈60 mAh g^−1^ at 4 A g^−1^ for a stable cycling life of up to 7500 cycles with negligible capacity decay (Figure 8d). To further improve the electrochemical performance of AIBs, considerable attention has been paid to optimizing the graphitic cathode, the electrolyte, and the aluminium as anode or anode/current collector.^77^, ^88^ Moreover, an aluminium–graphite dual‐ion battery containing an aluminium anode, a graphitic cathode, and 1 m LiPF_6_ EC–EMC–DMC electrolyte was also fabricated.^89^ Unlike the traditional AIB system, the aluminium in this system plays a critical role as substrate and reaction agent to form AlLi. As shown in **Figure** **9**a, the use of Al significantly decreases the risk of lithium dendrites and their related safety issues. In addition, the alloying/de‐alloying between Al and Li effectively improve the working voltage of the full cell compared to Al‐graphite using an Al‐based electrolyte (Figure 9b). The detailed half‐cell reactions at both negative and cathode electrodes are expressed as follows:

(8) $$\mathrm{Anode}:\;\;\mathrm{Al}+{\mathrm{Li}}^{+}+e^{-}↔\mathrm{AlLi}$$

(9) $$\mathrm{Cathode}:\;\;xC+{\mathrm{PF}}_{6}{}^{-}↔Cx{\mathrm{PF}}_{6}+e^{-}$$

(10) $$\mathrm{Total}:\;\;\mathrm{Al}+xC+{\mathrm{LiPF}}_{6}↔\mathrm{AlLi}+Cx{\mathrm{PF}}_{6}$$

The discharge product of the reaction is confirmed by XRD (Figure 9c). The cell exhibited a high reversible capacity of ≈100 mAh g^−1^ at 0.05 A g^−1^ and maintained a capacity retention of 88% after 200 charge‐discharge cycles at 0.2 A g^−1^ (Figure 9d). Furthermore, the electrochemical stability of aluminium as a current collector and anode was also evidenced in sodium‐based “dual‐ion” cells, which displayed stable reversible specific capacities of 15 mAh g^−1^ for 350 cycles in NaBF_4_ in EC/DEC and 55 mAh g^−1^ for 100 cycles in NaPF_6_ in EC/DEC.^90^

## 5 Conclusions and Perspectives

In summary, GICs for alkali ion‐based batteries will continue to hold great promise in energy‐related storage systems in the near future because of their unique chemical and physical properties, and the rich and excellent electrochemical activity of GICs. Graphite with a lithiation mechanism resulting in LiC_x_ suffers from limited electrochemical performance in particular, low capacity and poor rate capability). Owing to the comparatively high natural abundance, low cost, and many similar chemical/physical properties of Na and K to Li, many efforts have also been made to develop graphitic materials for SIBs and PIBs, similar to their LIB counterparts. Compared with the research on LIBs, the development history of SIBs and PIBs (especially PIBs) is quite short. It is very much expected that a great number of developed graphitic anode materials or promising ones undergoing development, as well as carbonaceous materials, for LIBs with high theoretical capacities, high surface area, high conductivity, and superb chemical stability would also be available for SIBs and PIBs. Apart from the development of carbonaceous materials for advanced metal ion batteries, the discovery of advanced electrolytes leading to the formation of ternary GICs (tGICs) with co‐intercalation of electrolyte solvents and alkali ions is also a promising and insightful topic for investigation in research on graphite, as well as other related carbonaceous materials.

Compared to cationic GICs, anionic GICs for metal ion batteries often employ different types of anions (e.g., PF_6_^−^, BF_4_^−^, TFSI^−^, ClO_4_^−^, CF_3_SO_3_^−^, (CF_3_SO_2_)_2_N^−^, and other fluoride ions) that can be electrochemically intercalated into graphite. It is undoubtedly recognized that there has been more impressive progress on and research interest in the cationic GICs than in the anionic GICs. Because anion intercalation into graphite often occurs at high potentials close to 5 V, retaining the stability of both the electrolyte solvent and the anions is a big challenge. Conventional commercial electrolyte solvents (e.g. EC, DEC, EMC, and DMC) are mainly subjected to oxidation (<4.6 V, vs. Li^+^/Li). ILs are a good candidate because of their non‐volatility, non‐flammability, high thermal and chemical stability, and wide electrochemical window. The high viscosity and high cost of ILs are critical issues, however, that should be addressed in the future. Compared to alkali‐based anionic GICs, aluminium‐graphite batteries have a narrow electrochemical window with an aluminium anode (vs. Li/Na/K). As expected, the combination of aluminium and alkali metal as anode or anode/collector could be a good choice for developing high‐energy Al/alkali‐metal‐graphite batteries with a wide range of electrolytes.

Moreover, the modification of graphite (e.g., expanded graphite, edge functionalization, heteroatom doping, computational calculations) is another potential way to change the energy barrier of the host structure and thus facilitate the intercalation/release of anions within a suitable electrochemical window. In order to gain more insight into the formation of the structure of the GICs and the electrolytes that participate in the electrochemical processes, in situ characterization techniques are also welcome. In addition, the fabrication of all‐graphite batteries with high capacity and long‐term cycling stability are also of great interest in the near future. Therefore, further development of GICs for metal (metal = Li, Na, K, Al) ion batteries is not only a great addition to the huge commercial success of lithiated graphite in LIBs, but also an effective way to develop diverse high‐energy batteries for stationary energy storage in the future.

## Conflict of Interest

The authors declare no conflict of interest.
